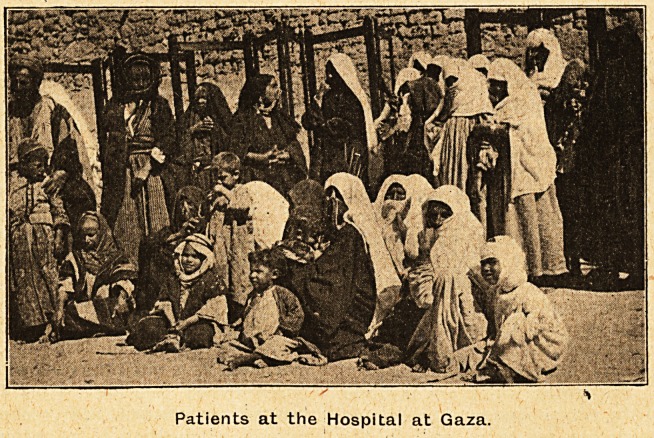# Work at Gaza

**Published:** 1917-12-15

**Authors:** 


### PALESTINE AND ITS HOSPITALS.

### Work at Gaza.

The British advance in Palestine has led the Church Missionary Society to issue an appeal for funds to re-open its hospital at Gaza. The war had interrupted or closed the work of the hospitals in Gaza and Jerusalem, and yet the record of their work before the war shows its extent; nor can the need at present be any less than it was.

The C.M.S. Hospital at Gaza.

The pleasant surroundings of the hospital at Gaza, which appear in the accompanying photograph, remind people in England that Philistia is a charming place in spring. One of the nurses on the hospital staff, Miss E. G. Williams, recently sent home an account of Gaza, on which, through the courtesy of the *Church Missionary Gleaner*, the following summary is based. The hospital serves a population of 30,000, most of whom are Moslems, and stands on the sea side of the town. It consists of a compound containing wards for men and for women, a theatre, a dispensary, and an out-patient department. The out-patients included Turkish officials, "peasants," and even the Bedouin, whose camps, being close to Gaza, were visited by the medical staff. Syrian girls were trained as probationer nurses.

Patients at the Hospital at Gaza.

Since the British advance, news has reached the Society that the olive groves of the city, which sheltered Turkish snipers, were heavily shelled during the second attack. Samson's Hill, the mound near the town, to which he is said to have carried the gates of Gaza, was turned into a fortress by the Turks. The hospital compound is reported to have escaped injury, and is now being used as a military hospital, where the Red Crescent, we understand, continues to fly. Of the three mosques in the town, one of which was originally the Crusaders' Church, two escaped injury. The third, a correspondent informs the Society, was used for military purposes, and was consequently shelled. The mud houses and the narrow mud streets, in which the bulk of the inhabitants live, seem to have been untouched, and the hospital is about to take up the work again in response to an urgent message from the military authorities.

The British Ophthalmic Hospital at Jerusalem was a very important institution before it was closed in consequence of the war. It used to treat annually 848 in-patients and no fewer that 6,060 out-patients.

What will its condition be on General Allenby's entry into the city, the official news of which comes as we write?

## Figures and Tables

**Figure f1:**
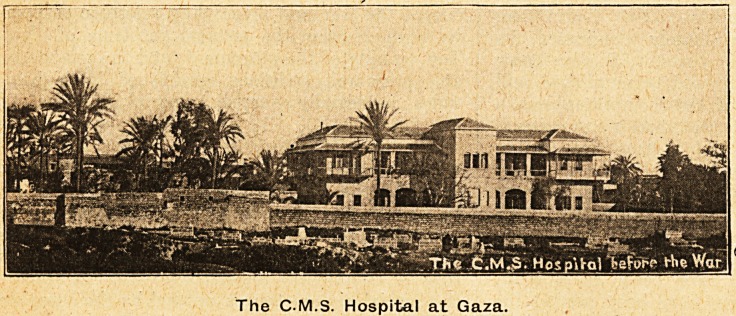


**Figure f2:**